# Supplementary material for: Medium spiny neurons activity reveals the discrete segregation of mouse dorsal striatum
Source: eLife. 2021 Feb 18;10:e60580. doi: 10.7554/eLife.60580 (PMC7924950; doi:10.7554/eLife.60580)
Supplement: Supplementary file 1. — (A) Featurization of the SWO of DCS-MSNs. Note that the number preceding the feature labels are the same as the ones showed in Figure 3. All values display means ± standard deviation. (B) Data set description. The three first rows are total values; the rest display the mean ± standard deviation. [file elife-60580-supp1.docx]

**Supplementary File 1. Additional data.**

**A.** Featurization of the SWO of DCS-MSNs. Note that the number preceding the feature labels are the same as the ones showed in figure 3. All values display means ± standard deviation.

**B.** Data set description. The three first rows are total values; the rest display the mean ± standard deviation.

| **Feature** | **Mean value** |
| --- | --- |
| 1. Average Vm in Up state (mV) | -54.44 ± 6.28 |
| 1. Up state standard deviation (mV) | 5.32 ± 1.36 |
| 1. Min Vm in Up state (mV) | -59.95 ± 6.23 |
| 1. Max Vm in Up state (mV) | -43.99 ± 6.20 |
| 1. Up state amplitude (mV) | 13.62 ± 4.10 |
| 1. Max of Up state derivative (dmV/dt) | 0.65 ± 0.14 |
| 1. Min of Up state derivative (dmV/dt) | -0.512 ± 0.12 |
| 1. Down to Up transition slope (mV/s x 10^2^) | 0.829 ± 0.25 |
| 1. Up to Down transition slope (mV/s x 10^2^) | 0.514 ± 0.13 |
| 1. Slope transition ratio | 1.61 ± 0.36 |
| 1. Number of peaks in Up state | 1.20 ± 0.15 |
| 1. Peak to peak in Up state (mV) | 20.74 ± 4.65 |
| 1. Up state length (ms) | 431.40 ± 71.50 |

**Supplementary File 1A.** **Featurization of the SWO of DCS-MSNs.**

| **Animals** | 45 |
| --- | --- |
| **Males** | 19 |
| **Females** | 26 |
| **Weight (gr)** | 33.54±1.37 |
| **Male weight (gr)** | 30.04±1.58 |
| **Female weight (gr)** | 37.58±1.94 |
| **Age (weeks)** | 24.66±1.31 |
| **Male age (weeks)** | 22.52±1.79 |
| **Female age (weeks)** | 27.26±1.84 |
| **Cells/animal** | 4.38±0.49 |
| **Cells/male** | 4±0.92 |
| **Cells/female** | 4.69±0.39 |

**Supplementary File 1B.** **Data set description.**
